# Relationship between serum irisin, glycemic indices, and renal function in type 2 diabetic patients

**DOI:** 10.15171/jrip.2017.17

**Published:** 2016-11-20

**Authors:** Leila Mahmoodnia, Maryam Sadoughi, Ali Ahmadi, Marzieh Kafeshani

**Affiliations:** ^1^Department of Internal Medicine, Shahrekord University of Medical Sciences, Shahrekord, Iran; ^2^Department of Epidemiology and Biostatistics, Shahrekord University of Medical Sciences, Shahrekord, Iran; ^3^School of Nutrition & Food Science, Isfahan University of Medical Sciences, Isfahan, Iran

**Keywords:** Irisin, Type 2 diabetic patients, Body mass index

## Abstract

**Introduction:** Irisin is a novel peptide that plays notable role in human and animal biology and physiology. It has been reported that irisin may improve insulin resistance and related disturbances.

**Objectives:** The aim of this investigation was to assess the relationship between serum irisin, glycemic indices, and renal function in diabetic subjects.

**Patients and Methods:** In this cross-sectional study, a total of 102 type 2 diabetes mellitus (T2DM) patients were recruited. Blood biochemical parameters, including fasting plasma sugar (FBS), glycosylated hemoglobin (HbA_1C_), serum uric acid (sUA), creatinine concentration and glomerular filtration rate (GFR) were measured. All statistical analysis was performed with SPSS 16.0.

**Results:** There was a positive correlation between irisin and age (*P*=0.05, r=0.19) and a negative correlation between irisin and body mass index (BMI) (*P*=0.01, r=-0.25) was detected. There was a significant difference of serum irisin level between patients with normal and abnormal FBS too.

**Conclusion:** In this study we found, irisin concentration was increased with age, decreased with BMI, and it was higher in subject with abnormal FBS. Thus further research is needed to provide inclusive understanding of irisin associated physiological effects and possible implications in clinical conditions.

**Implication for health policy/practice/research/medical education:**

In a study on 102 type 2 diabetic patients, we found, irisin concentration was increased with age, decreased with BMI, and it was higher in subject with abnormal FBS.

## Introduction

The worldwide prevalence of diabetes was estimated 382 million people by the International Diabetes Federation in 2013. Diabetes mellitus is a complex public health concern that is related with various micro-vascular (neuropathy, nephropathy, and retinopathy) and macro-vascular (peripheral arterial disease, coronary artery disease, and stroke) complications. These complications cause vast disability, morbidity, and mortality, and even low quality of life. There are different factors that influence on the risk of developing diabetes and its complication (1).

Recently, researchers have shown an increased interest in association between irisin level and diseases. Irisin is a novel peptide that plays notable role in human and animal biology and physiology. It has been reported that irisin may improve body weight states and insulin resistance. Irisin was first introduced as exercise-induced myokines by Boström et al in 2012, but other studies showed it also is an adipokine that secreted by subcutaneous adipocytes and affected by exercise and nutritional factors (2). In brief, Boström et al demonstrated that exercise enhances levels of peroxisome proliferator-activated receptor-γ coactivator-1α (PGC*-*1α) in the muscle thus the expression of the type I membrane precursor protein fibronectin-type III domain-containing 5 (FNDC5) was induced. Then irisin was produced by attaching N-terminal fibronectin III (FNIII)-like domain to a flexible C-terminal. Irisin stimulated the expression of brown adipose tissue (BAT) related genes such as uncoupling protein 1 (UCP1, highly expressed in BAT and a marker of browning), partially through increased PPAR–α and other incompletely understood method (3). Lesser circulating irisin was related with the insulin resistance, diabetes (4,5), chronic kidney disease (6,7) and other disease (8). The results of studies on irisin and glucose homeostasis have been controversial. Some studies established that serum irisin was significantly negatively correlated with hemoglobin A_1C_, fasting blood glucose, 2 hours plasma glucose and homeostasis model assessment of insulin resistance (HOMA-IR) and other studies found positive correlation (9). Studies regarding relationship between serum irisin and renal functional markers are scares however these researches suggested that irisin level might be associated with renal function in humans.

## Objectives

According to the limited studies and the controversy about scientific evidence, there is a need for further research to identify the relationship between irisin, and clinical and biochemical markers of various chronic diseases. The main aim of this investigation was to assess the relationship between serum irisin, glycemic indices, and renal function in diabetic individuals.

## Patients and Methods

### Patients

In this cross-sectional study, a total of 102 type 2 diabetes mellitus (T2DM) patients were recruited from Imam-Ali clinic, Shahrekord, Iran. In the present study, T2DM was defined using the American Diabetes Association (ADA) criteria. Standard questionnaire was used for assessing information, including age, sex, disease history, anti-diabetic and anti-hypertensive drugs. Diabetic patients on dialysis, patients with kidney transplants**,** and mentally retarded patients who were not able to give informed consent were excluded. Additionally patients with malignancy and active or chronic infections were excluded.

### Ethical issues

The research adhered to the principles of the Declaration of Helsinki; the purpose and design of study were explained for all subjects and they completed an informed consent form. Ethical Committee of Shahrekord University of Medical Science approved the protocol (#IR.SKUMS.REC 1394.165).

### Biochemical assessments

Blood biochemical parameters, including fasting plasma sugar (FBS), serum uric acid (sUA) and creatinine (Cr)concentration were measured by commercially available enzyme assay kits (Pars Azmon kit, Iran). HbA_1C_ was measured with chromatography using Biosystem kit (Spain). Glomerular filtration rate (GFR) was estimated from the results of blood Cr test and by the Modification of Diet in Renal Disease (MDRD) equation. Circulating irisin was quantified using a commercial ELISA kit (BioVendor – Laboratorni Medicina, Czech Republic).

### Clinical measurements

Blood pressure (BP) was determined in a seated position where their arm sustained at heart level, after 5 minute rest. BP was noted as three sequential amounts at intervals of 30 seconds. The mean of the three BP measurements was utilized in the analysis.

### Statistical analysis

The normality of data was tested by using Kolmogorov-Smirnov test. The nonparametric statistical methods were used to analyses the numerical data because the data had no normal distribution. Parametric variables were extracted as mean ± standard deviation (SD). Categorical data were stated as percent and compared by χ^2^ test. Median and quartile were used to describe nonparametric data. Mann–Whitney U rank test was used to compare differences between groups. Kruskal–Wallis one-way analysis of variance test was used to compare irisin between different levels of body mass index (BMI). Spearman’s correlation coefficient and partial correlation tests were applied to evaluate the correlation between nonparametric variables. The data were analyzed by SPSS version 16. In this study, *P* value below 0.05 was respected as statistically significant for all tests.

## Results

Table 1 illustrates some of the main characteristics of the subjects. The mean age was 61.25 ± 11.7 years. The majority of subjects were women (68% of total), and duration of diabetes was 7.64 years.

**Table 1:** Basic characteristics of diabetic subjects (n = 103)

	**Mean or median** ^c^	**SD or range**
Age (years)	61.25	11.70
BMI (kg/m^2^)	29.32	5.48
Male (%)	32	
Types of drug (%)		
Insulin	10.9	-
Oral hypoglycemic agents	89.1	-
Duration of diabetes (years)	7.64	0.61
FBS (mg/dL)	140	109-183
HbA_1c_ (%)	7.50	6.10-9.00
sUA (mg/dL)	4.70	3.95-5.60
Cr (mg/dL)	0.90	0.80-1.00
eGFR^a^ (mL/min/1.73 m^2^)	83.70	64.45-100.83
GFR (MDRD)^b^ (cc/min)	72.07	63.39-83.21
Irisin (ng/dL)	2.60	1.80-3.40

Abbreviations: BMI, body mass index; FBS, fasting blood sugar; HbA_1c_, glycosylated hemoglobin; sUA, serum uric acid; Cr, creatinine. ^a^eGFR (estimated glomerular filtration rate) calculated from the results of blood creatinine, age, body size and gender. ^b^ GFR (MDRD), calculated from the Modification of Diet in Renal Disease (MDRD) equation. Data for categorical variables are presented as percentages. ^c^Parametric and non-parametric variables were extracted as mean ± SD and median, quartile respectively.

The results of the correlational analysis are presented in Table 2. In this study a positive correlation between serum irisin and age (*P* = 0.05, r = 0.19) and a negative correlation between irisin and BMI was detected (*P* = 0.01, r = -0.25). Accordingly a positive correlation between age and serum Cr (*P* = 0.02, r = 0.23) was detected.

**Table 2:** Correlation between serum irisin, glycemic indices, and renal function in diabetic patients

	**Irisin**	**Age**	**sUA **	**sCr**	**eGFR**	**GFR**	**FBS**	**HbA**_1c_	**HTN**	**BMI**
Irisin		0.19^a^	0.01	-0.01	-0.14	0.01	-0.18^b^	-0.09	-0.07	-0.25^a^
Age			0.1	0.23^a^	-0.66^a^	-0.4^a^	-0.07	-0.09	-0.13	-0.18
sUA				0.44^a^	-0.14	-0.37^a^	-0.16	-0.27^a^	0.11	0.24^a^
Cr					-0.46^a^	-0.74^a^	-0.07	-0.01	-0.07	0.03
GFR (mL/min/1.73 m^2^)						0.72^a^	0.05	-0.08	0.19	0.54^a^
GFR (MDRD) (cc/min)							0.08	0.01	0.29^a^	0.05
FBS								0.55^a^	0.16	-0.1
HbA_1c_									0.16	-0.18

Abbreviations: BMI, body mass index; FBS, fasting blood sugar; HbA_1c_, glycosylated hemoglobin; sUA, serum uric acid; sCr, serum creatinine. eGFR, estimated glomerular filtration rate; HTN, hypertension; MDRD, Modification of Diet in Renal Disease. ^a^ P < 0.05 is significant; ^b^ is marginally significant.

**Table 3:** Compare circulating irisin between diabetic subjects with normal and abnormal biochemical characteristics

	**Normal**			**Abnormal**			***P*** ** value**
	**Percent**	**Mean (ng/dL)**	**SD**	**Percent**	**Mean (ng/dL)**	**SD**	
sUA (mg/dL)	85.1	2.98	0.22	14.9	2.86	0.21	0.71
Cr (mg/dL)	95	2.86	0.15	5	3.2	0.49	0.37
eGFR (mL/min/1.73 m^2^)	39.6	2.67	1.18	60.4	3.10	1.81	0.29
GFR (MDRD)	10.9	2.88	1.84	89.1	2.94	1.58	0.37
FBS (mg/dL)	89.1	2.81	1.55	10.9	3.92	1.73	0.009
HbA_1c_ (%)	43.6	2.87	1.37	56.4	2.98	1.77	0.84

Abbreviations: FBS, fasting blood sugar; HbA_1c_, glycosylated hemoglobin; sUA, serum uric acid; Cr, creatinine; eGFR, estimated glomerular filtration rate; MDRD, Modification of Diet in Renal Disease.

**Table 4:** Association of circulating irisin with demographic parameters and BMI levels in diabetic subjects

	**Mean (ng/dL)**	**SD**	***P*** ** value**
Sex			0.58
Female	2.82	0.16	
Male	3.18	0.35	
Age			0.52
≤45	2.42	0.83	
>45	2.97	1.64	
BMI (kg/m^2^)			0.05^a^
≤ 18.5	6.23	4.2	
18.5-24.9	3.22	1.45	
25-24.9	3.03	1.52	
>30	2.49	0.96	
Types of drug			0.48
Insulin	2.95	1.58	
OHA	2.8	1.87	

Abbreviations: BMI, body mass index; OHA, oral hypoglycemic agents. ^a^ *P* < 0.5 is significant.

Spearman’s correlation analysis was performed to study the correlation between variables. The association between circulating irisin and biochemical and demographic parameters was shown in Tables 3 and 4. Data from both tables shows that, the majority of individuals had normal FBS (mg/dL), HbA_1C_ (%), and GFR, however sUA (mg/dL) and sCr (mg/dL) in the most of them were abnormal. There was a significant difference in serum irisin level between patients with normal and abnormal FBS (mg/dL), but there was no significant differences in irisin level between subjects with normal and abnormal other biochemical parameters. Also there was no significant difference between men and women and patients who taking insulin or oral hypoglycemic agents.

## Discussion

The present study was designed to determine relationship between serum irisin, glycemic indices, and renal function in diabetic patients. The results of this study indicated a positive correlation between irisin and age and a negative correlation between irisin and BMI. We also found a marginally negative correlation between irisin and FBS. However no significant correlation between circulating irisin and HbA_1c_ was detected. There was a significant difference in serum irisin level between the subject with normal and abnormal FBS, so it can be concluded that circulating irisin was higher in diabetic subjects with uncontrolled FBS. These results are consistent with other studies. Huh et al found circulating irisin concentrations were positively correlated with FBS (5) and Mehrabian et al determined serum irisin level correlated positively with insulin levels and FBS in normal weight obese subjects (10). Also Liu et al demonstrated the positive correlation between circulating irisin and FBS in non-obese, non-diabetic individuals. These results differ from some published studies (7). Zhang et al and Du et al in two different meta-analysis established that circulating irisin concentrations were significantly lower in patients with T2DM (4,11). Sanchis-Gomar et al reported positive correlation between irisin level and HbA_1c_ in T2DM patients with and without obesity (12). While, the majority of subjects were obese or over weight hence one explanation for our result is “irisin resistance” in obese people as same as leptin and insulin resistance (13). Our findings revealed that no correlation between irisin and renal function tests and indices such as sUA, Cr, and GFR was detected. Also no significant difference between the subjects with normal and abnormal renal function markers was seen. The findings of the current study do not support the previous research (6,7), while, Wen et al established that plasma irisin levels were significantly decreased in chronic kidney disease (CKD) patients (6,7). Accordingly, Liu et al found that circulating irisin was significantly decreased in diabetic patients with renal insufficiency compared to T2DM patients with normal renal function. They found that circulating irisin was independently associated with GFR (7). Yang et al determined that high serum irisin level was associated with reduced risk of chronic kidney disease (CKD) (14). A possible explanation for this inconsistency may be due to studied different populations.

## Conclusion

This study presented the relationship between serum irisin, glycemic indices, and renal function in diabetic subjects. This research has shown a positive correlation between irisin and age and a negative correlation between irisin and BMI (*P* = 0.01, r = -0.25). We also showed a significant difference in serum irisin level between the subjects with normal and abnormal FBS. In general, it seems that serum irisin concentration was increased with age and decreased with BMI and it was higher in subject with abnormal FBS. However, further research is suggested to provide inclusive understanding of irisin associated physiological effects and possible implications in clinical conditions.

## Limitations of the study

Low proportion of patients is a limitation of our study.

## Authors’ contribution

All authors participated to design of the study. LM managed the research. MS performed the investigation. AA and MK analyzed the data. MK prepared the manuscript. All authors read, revised and approved the manuscript.

## Conflicts of interest

The authors declare that they have no competing interests.

## Ethical considerations

Ethical issues (including plagiarism, data fabrication, double publication) have been completely observed by the authors.

## Funding/ Support

This study was extracted from MD thesis of Maryam Sadoughi (# 1252) and supported by the Vice-Chancellery for Research and Technology of Shahrekord University of Medical Sciences.
